# Supplementary material for: HDAC6 Enhances Endoglin Expression through Deacetylation of Transcription Factor SP1, Potentiating BMP9-Induced Angiogenesis
Source: Cells. 2024 Mar 11;13(6):490. doi: 10.3390/cells13060490 (PMC10969049; doi:10.3390/cells13060490)
Supplement: Supplementary file 1 [file cells-13-00490-s001.zip › Figure S1.pdf]

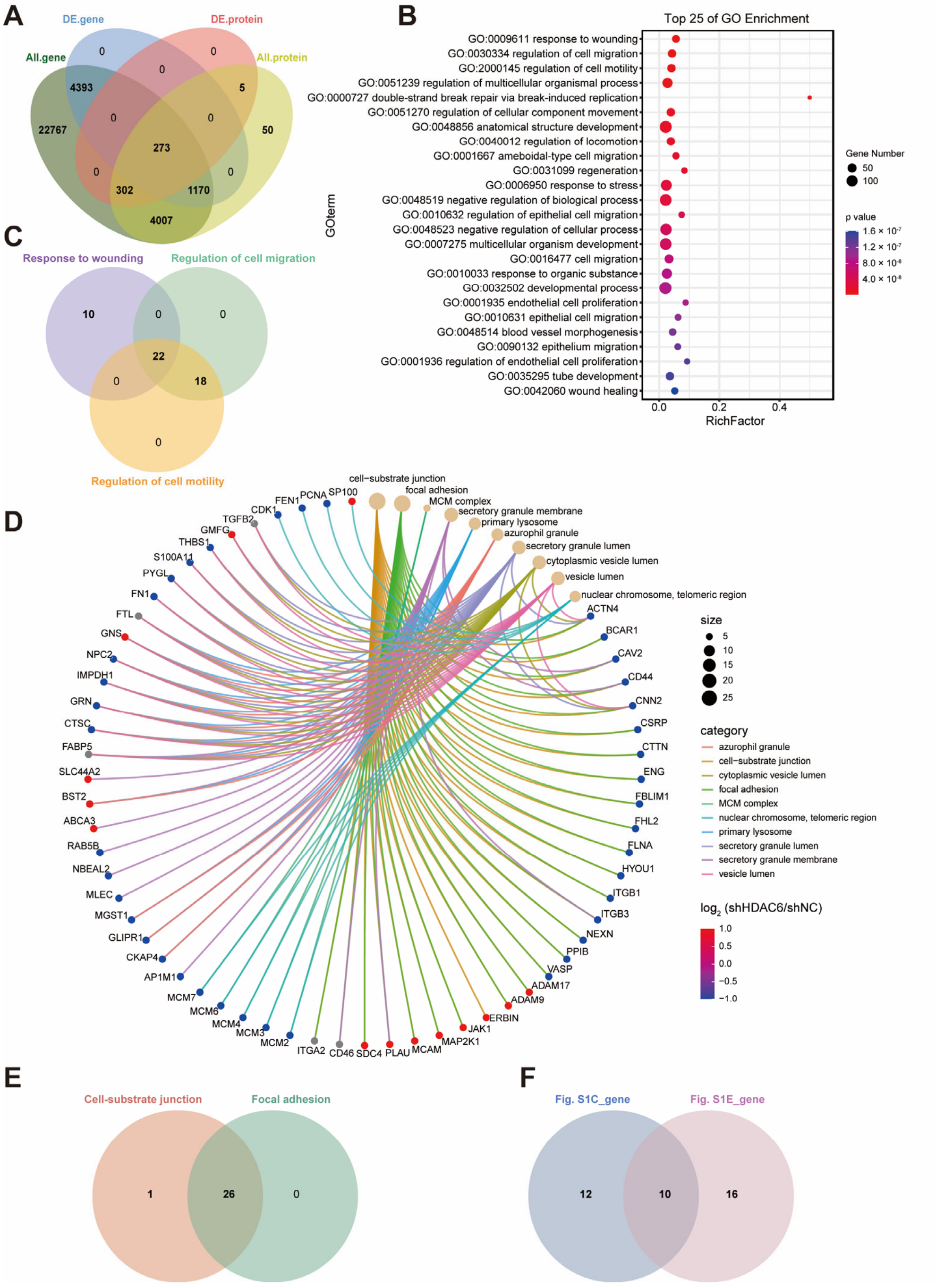

Figure S1. Integrated bioinformatics analysis of differentially expressed genes following HDAC6 knockdown in HUVECs. (**A**), Venn diagram displaying the counts of differentially expressed (DE) mRNAs and proteins, including their overlap, due to HDAC6 knockdown. (**B**,**C**), GO enrichment analysis illustrates the significant biological processes enriched as a result of HDAC6 knockdown in HUVECs and the corresponding Venn diagram displaying the counts of overlapping genes from the top 3 categories. (**D**,**E**), Network analysis diagram illustrating the cellular components affected by HDAC6 knockdown in HUVECs and the corresponding Venn diagram displaying the counts of overlapping genes from the cell-substrate junction and focal adhesion categories. (**F**), Venn diagram demonstrating the intersection of genes identified in Figure S1C,D.
