# Supplementary material for: HDAC6 Enhances Endoglin Expression through Deacetylation of Transcription Factor SP1, Potentiating BMP9-Induced Angiogenesis
Source: Cells. 2024 Mar 11;13(6):490. doi: 10.3390/cells13060490 (PMC10969049; doi:10.3390/cells13060490)
Supplement: Supplementary file 1 [file cells-13-00490-s001.zip › Supplementary Tables.pdf]

Table. S1 Antibodies used in this manuscript

Antibodies	Source	Identifier
Mouse anti-Acetyl Lysine	Abcam	Cat# ab22550
Rabbit anti-Acetylated-Lysine	Cell Signaling Technology	Cat# 9441S
Mouse anti-HA	ThermoFisher Scientific	Cat# 26183
Mouse anti-FLAG	Cell Signaling Technology	Cat# 8146
Mouse anti-alpha Tubulin (acetyl K40)	Abcam	Cat# ab24610
Rabbit anti-Tubulin	Abcam	Cat# ab52866
Rabbit anti-HDAC6 (D2E5)	Cell Signaling Technology	Cat# 7558
Mouse anti-HDAC6 (OTI3E7)	ThermoFisher Scientific	Cat# MA525359
Rabbit anti-ENG	Abcam	Cat# ab252345
Mouse anti-GAPDH	Proteintech	Cat# 60004
Rabbit anti-SMAD1/5/9 (phospho S463 + S465 + S467)	Abcam	Cat# ab92698
Rabbit anti-SMAD1/5/9	Abcam	Cat# ab300164
Rabbit anti-Smad2/3 (phospho T8)	Abcam	Cat# ab254407
Rabbit anti-Smad2/3	Abcam	Cat# ab202445
Rabbit anti-SP1	Abcam	Cat# ab231778
Goat Anti-Rabbit IgG	Beyotime	Cat# A0208
Goat Anti-Mouse IgG	Beyotime	Cat# A0216

Table. S2 qPCR primers used in this manuscript

Gene Name	Forward Primer Sequence (5' -> 3')	Reverse Primer Sequence (5' -> 3')
HDAC6	AAGAAGACCTAATCGTGGGACT	GCTGTGAACCAACATCAGCTC
ENG	GCCTCCCCTACTGTCCTTTG	CGCATGTCCCAGCTATCGTC
SP1	TGGCAGCAGTACCAATGGC	CCAGGTAGTCCTGTCAGAACTT
Actin	CATGTACGTTGCTATCCAGGC	CTCCTTAATGTCACGCACGAT
